# Scale up of Transmembrane NADH Oxidation in Synthetic Giant Vesicles

**DOI:** 10.1021/acs.bioconjchem.1c00096

**Published:** 2021-04-27

**Authors:** MinHui Wang, André Weber, Roland Hartig, Yiran Zheng, Dorothee Krafft, Tanja Vidaković-Koch, Werner Zuschratter, Ivan Ivanov, Kai Sundmacher

**Affiliations:** † Process Systems Engineering, Max Planck Institute for Dynamics of Complex Technical Systems, Sandtorstrasse 1, 39106 Magdeburg, Germany; ‡ Combinatorial Neuroimaging Core Facility, Leibniz Institute for Neurobiology, Brenneckestrasse 6, 39118 Magdeburg, Germany; § Institute of Molecular and Clinical Immunology, Otto-von-Guericke University Magdeburg, Leipziger Strasse 44, 39120 Magdeburg, Germany; ∥ Electrochemical Energy Conversion, Max Planck Institute for Dynamics of Complex Technical Systems, Sandtorstrasse 1, 39106 Magdeburg, Germany; ∇ Department of Process Systems Engineering, Otto-von-Guericke University Magdeburg, Universitätsplatz 2, 39106 Magdeburg, Germany

## Abstract

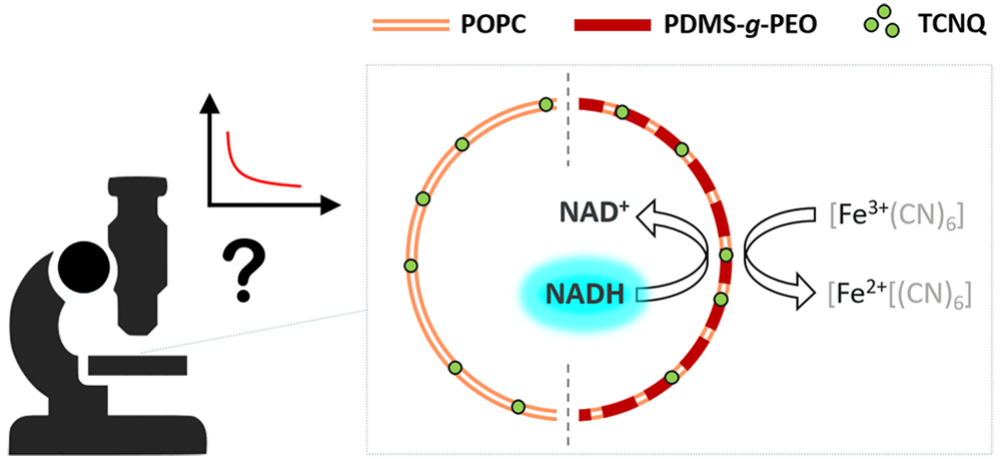

The transfer of electrons across and along biological membranes drives the cellular energetics. In the context of artificial cells, it can be mimicked by minimal means, while using synthetic alternatives of the phospholipid bilayer and the electron-transducing proteins. Furthermore, the scaling up to biologically relevant and optically accessible dimensions may provide further insight and allow assessment of individual events but has been rarely attempted so far. Here, we visualized the mediated transmembrane oxidation of encapsulated NADH in giant unilamellar vesicles via confocal laser scanning and time-correlated single photon counting wide-field microscopy. To this end, we first augmented phospholipid membranes with an amphiphilic copolymer in order to check its influence on the oxidation kinetics spectrophotometrically. Then, we scaled up the compartments and followed the process microscopically.

Membrane phenomena play a crucial role in living cells and, among other functions, enable out-of-equilibrium states, which drive the cellular metabolism. E.g., the proton gradient generated by oxidation of NADH and other reduced substrates in the electron transport chain drives ATP synthesis.^1^ In the origin-of-life conundrum, the universality of these pathways contradicts the complexity of the energy-transducing machinery, which is addressed in minimal configurations of simpler and plausible catalysts.^2^ On the other side, bottom-up synthetic biology is not burdened by evolutionary constraints and offers the possibility for reproduction of fundamental mechanisms by the use of highly evolved molecules or even fully synthetic alternatives.^3^ In line with these motivations, we have assembled a minimal oxidative phosphorylation system in polymersomes^4^ and transmembrane cofactor oxidation in liposomes, mediated by the electron shuttle tetracyanoquinodimethane (TCNQ).^5^ Regarding the latter, electron transfer across vesicle bilayers has been almost exclusively studied in nanocompartments,^6^ which restricts the assessment to bulk methods and may necessitate additional controls, e.g., to ensure compartment integrity. Thus, there is an incentive to employ objects, compatible with optical methods, in order to provide straightforward confirmation and access individual events. Furthermore, microcompartments such as giant unilamellar vesicles (GUVs) are established workhorses for mimicking cellular functions due to the structural and dimensional similarity to modern cells.^7^

In the present work, we spectroscopically tested the influence of the synthetic amphiphile poly(dimethylsiloxane)-*graft*-poly(ethylene oxide) (PDMS-*g*-PEO) on the interfacial oxidation of encapsulated NADH at the nanoscale, seeking improved performance. Next, we scaled up the transmembrane setup to micrometer vesicles. During the microscopic monitoring of the NADH fluorescence, we faced a bleaching issue due to the low quantum efficiency of NADH (1.9% in aqueous solution^8^). This shortcoming was amplified at high intensity or point scanning illumination, and it is expected to appear not only in minimal systems but also in living cells, provided the established role of NADH as intrinsic bioenergetic marker,^9,10^ and altogether underlines the importance of sensitive low-noise imaging techniques for long-term monitoring of NADH kinetics.

Liposomes are simplified membrane models, which use the building blocks of living cells but lack sugar and protein ornaments. With respect to cellular mimicking, there is a parallel search for versatile and easily obtainable models with alternative chemistry that can provide favorable attributes for application.^11^ Polymer vesicles (polymersomes) typically have enhanced stability and lower permeability, while the trade-offs between natural and synthetic membranes can be alleviated by the use of hybrid systems.^12^ The latter approach is particularly relevant for the reconstitution of complex proteins when the supramolecular polymer arrangement does not correspond well to the natural phospholipid environment. PDMS-*g*-PEO, however, forms layers of similar thickness to lipid membranes and accommodates the bacterial proton pump cytochrome *bo*_3_ ubiquinol oxidase with fully retained activity.^4^ In addition to the matching dimensions, the protein compatibility is associated with the 20-fold higher fluidity of the latter polymer membrane, compared to the canonical poly(butadiene)-*block*-poly(ethylene oxide) (PBd-*b*-PEO) (4.1 ± 0.9 vs 0.22 ± 0.06 μm^2^ s^–1^).^13,14^ Since the diffusion of artificial mediators in bilayers is rate-limiting for the electron transfer,^15^ we hypothesized that the increased fluidity of PDMS-*g*-PEO (in the order of natural lipids, e.g., 11.3 ± 1.5 μm^2^ s^–1^ for soy phosphatidylcholine^16^) will qualify it as a suitable interface for transmembrane NADH oxidation by embedded TCNQ. Therefore, we first spectrophotometrically assessed the transmembrane electron transfer in ∼150 nm large unilamellar vesicles (LUVs) made of different amphiphiles. Thereby, in addition to the pure polymer, we tested hybrid membranes with different molar ratios of PDMS-*g*-PEO and 1-palmitoyl-2-oleoylphosphatidylcholine (POPC) and benchmarked them against lipid vesicles.^5^

The extrusion resulted in uniform size distributions (Figure S1), matching the pore size of the filter (<200 nm), and the polydispersity index was lower than 0.2. There was an overall decrease in the hydrodynamic diameter with an increasing polymer content, which may be associated with lower lysis tension,^17^ but since the extrusion was done manually and the applied pressure was not controlled, we did not investigate this further. Similar stochastic dependence on the polymer content was shown for PBd-*b*-PEO/POPC blends.^18^ TCNQ incorporation on the other side showed no significant effect on the LUV size.

For the spectrophotometric assessment of the transmembrane electron transfer in different membranes, we first performed the following control experiments for each composition in triplicates: blank vesicles, vesicles with membrane-incorporated TCNQ, and vesicles with encapsulated NADH in the presence of 400 μM ferricyanide but lacking the mediator (examples in Figures 1a and S2). Thereby, the first two controls served to validate the baseline from background fluorescence. The third control without the mediator assessed the physical separation between NADH and the external oxidant. It also allowed discriminating the initial transmembrane kinetics from the oxidation of small amounts of nonencapsulated NADH. The NADH fluorescence alone decreased by ∼10% within 3 h, but the kinetics in the presence of TCNQ was not corrected by this decay because it was not clear whether it was caused by degradation or photobleaching and if the resulting products could participate in the redox process.

**Figure 1:**
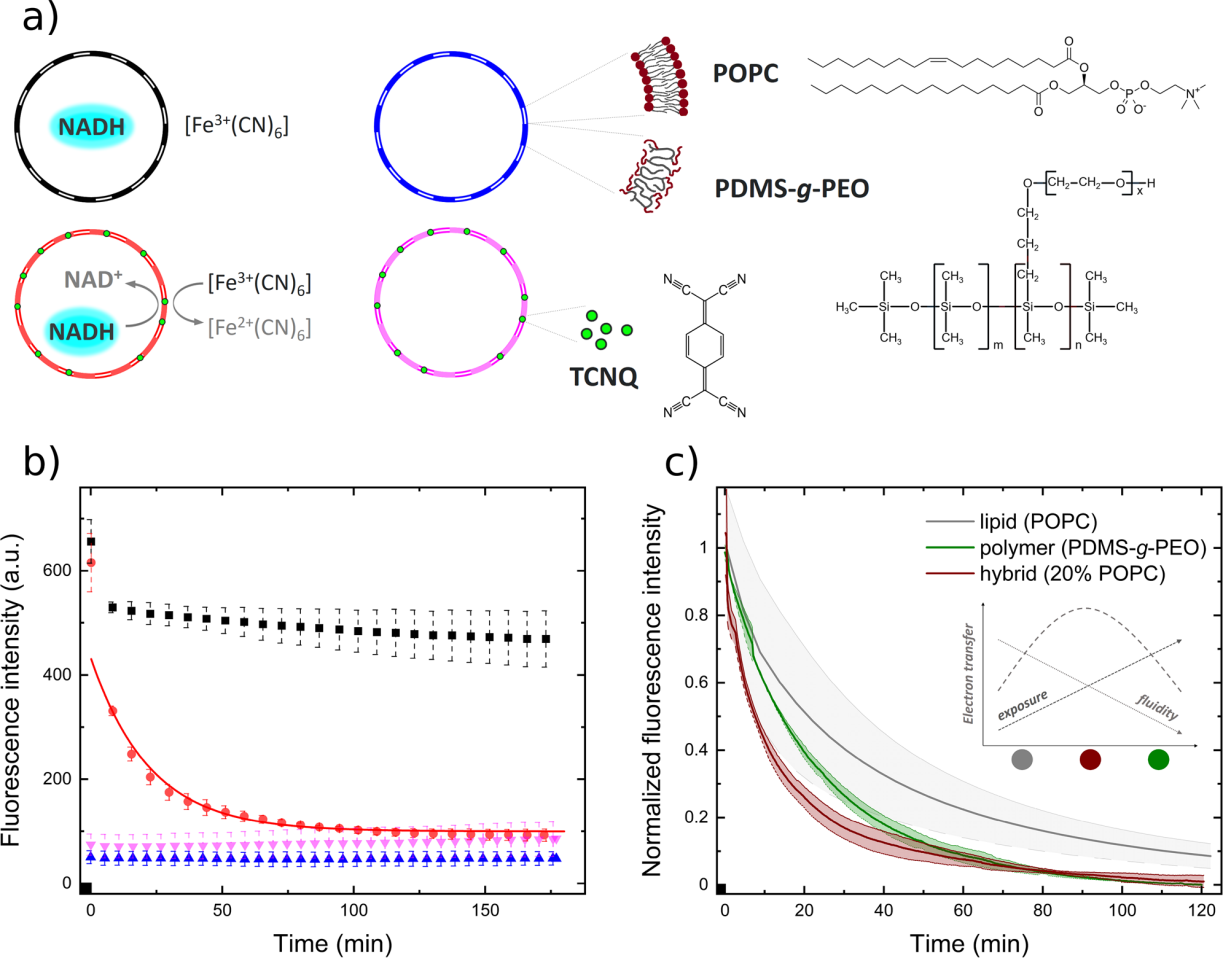
a) Schematic representations of the experiments shown in b) incl. chemical structures of the amphiphiles and the mediator. Color code corresponds to the respective traces. b) Spectrophotometric fluorescence profiles of hybrid vesicles composed of 20 mol % POPC/80 mol % PDMS-*g*-PEO with varying inner and outer membrane compositions. c) Normalized fluorescence profiles of NADH encapsulated in different types of LUVs with embedded TCNQ upon addition of 400 μm outer ferricyanide. The schematic inset shows a possible interpretation of the interplay between membrane order and fluidity.

The transmembrane electron transfer proceeded in vesicles with membrane-incorporated TCNQ and encapsulated NADH in the presence of 400 μM external ferricyanide (Figure 1a). We simplified the kinetic analysis^5^ by fitting a single exponential decay curve to obtain a lumped rate constant (Figure S2). The fitting protocol excluded the initial steep drop originating from the oxidation of small amounts of nonencapsulated NADH, which, in turn, may have led to a slight underestimation of the rates. Altogether, the hybrids containing 20 mol % POPC surpassed the other membranes with a rate constant of 7.4 × 10^–4^ s^–1^, followed by the pure polymer (5.7 × 10^–4^ s^–1^), and were nearly two times faster than pure POPC (the respective profiles, normalized to the starting fluorescence value and after extraction of the baseline, are shown in Figure 1b). We do not expect deviations in the LUV concentrations for different amphiphiles, because we followed identical preparation protocols.

In the case of the 50 mol % polymer/lipid blend, the ferricyanide control lacking mediator exhibited exponential decay, similar to the actual test with TCNQ, while the rest of the compositions showed a fairly linear response (Figure S2). Although bleaching and degradation may also proceed exponentially, this suggested possibly compromised compartment integrity. Therefore, the 50 mol % blend was excluded from further tests. In the case of hybrid LUVs containing ≥30 mol % POPC, the potential leakage can be ascribed to increased phase separation, as observed previously,^19^ which may cause multiple membrane defects at the phase junctions. In parallel, above 30 mol % POPC the demixing of polymer and lipid could result in distinct vesicle populations, which are not discernible in the present nanoscale experiments. Therefore, the membrane containing 80 mol % POPC was disregarded for further tests too, although it did not exhibit dubious behavior in the control experiments.

Considering a potential oxidant leak, the membrane integrity of the hybrids containing 20 mol % POPC was further tested by prolonged incubation in concentrated ferricyanide in order to rule out its penetration to the compartment interior. The oxidant was added to the vesicle suspensions in 50-fold concentration compared to the standard experimental conditions, and after overnight incubation, it was removed by gel filtration. No characteristic absorption at 420 nm was detected (Figure S3), and the size of the vesicles remained unchanged. While hybrid LUVs containing 30 mol % POPC previously exhibited nanodomains and enhanced proton permeability to a certain extent, full demixing to separate polymer and lipid populations was never observed via cryo electron microscopy.^16^ The lower amount of lipid in the present case suggests its more uniform distribution in the PDMS-*g*-PEO monolayer, hence we consider the hybrid vesicles homogeneous, while virtually impermeable to larger molecules like NADH and ferricyanide. The hybrid membrane containing 20 mol % POPC and 80 mol % PDMS-*g*-PEO accommodates both membrane integrity and enhanced electron transfer. The origin of the latter merit cannot be correlated with the membrane fluidity because hybrid and polymer membranes exhibited lower lateral diffusion coefficients in fluorescence recovery after photobleaching (FRAP) experiments.^16^ However, the increasing polymer content was also associated with a growing membrane disorder, evidenced by the bilayer probe Laurdan. Thus, we suggest that the optimal activity of hybrids originates from the trade-off between the mobility of the hydrophobic mediator and its exposure to the polar ferricyanide (and NADH), see the inset in Figure 1b. A similar effect of more pronounced quenching of photoexcited chlorophyll by ferricyanide has been ascribed to perturbation of the polar/nonpolar border and display of the chlorin ring to the water phase upon addition of cholesterol.^20^

Electroformation or electroswelling^21^ is one of the most widely used techniques for making micrometer vesicles due to the simple experimental setup, short duration, and attained quality (unilamellarity) of the membrane.^7^ Although tailored protocols for the growth of GUVs under physiological conditions exist,^22,23^ electroformation with high salt is generally considered difficult. Therefore, we modified the composition of the NADH solution and formed an ample amount of 10–30 μm hybrid and lipid GUVs in the presence of 1 mM NADH and sucrose (Figure S4). Higher NADH concentration or the presence of buffer resulted in lower GUV yield and diameter. Dilution of the resulting suspension with isosmotic glucose settled the GUVs to the bottom of the observation slide and provided sufficient contrast between the lumen and the exterior so that the separate vesicles could be easily identified by conventional epifluorescence microscopy.

Addition of 10 mM ferricyanide significantly decreased the blue fluorescence throughout the sample (Figure S4), whereby the nonencapsulated cofactor was oxidized first. A small portion of the compartments remained unaffected, which was ascribed to the presence of multivesicular vesicles and the potentially nonuniform distribution of TCNQ. Overall, the kinetics of transmembrane oxidation could not be resolved due to the severe bleaching of NADH (the weak intrinsic fluorescence disappeared even after prolonged focusing over tens of seconds). Therefore, the utility of epifluorescence microscopy with a standard camera in the present approach remained in the initial assessment of successful GUV formation and NADH encapsulation.

Confocal laser scanning microscopy (CLSM) is an established technique to observe membrane phenomena in GUVs, and we next used it to follow the NADH oxidation. The hybrid GUVs did not exhibit membrane protrusions, and the encapsulation was more uniform in comparison to pure POPC (Figure 2). The formation of buds and tubes in lipid bilayers is due to the excess area and the spontaneous curvature, which, in turn, is affected by solute asymmetry^24^ among other factors. Different salt and sugar concentrations across the membrane are present in the current system as well, but the predominant component of hybrids (i.e., PDMS-*g*-PEO) self-assembles into a soft monolayer, which appears to be less responsive to the unbalanced conditions. On the other side, the low encapsulation efficiency of large and charged molecules is a known drawback of both the spontaneous and the electrically assisted swelling of lipid films^7^ (note the absence of a cofactor in lipid GUVs with excess surface). In the case of hybrids though, NADH evidently better penetrates the amphiphile film during the formation, possibly due to less organized multilayers. In both types of GUVs, the intensity of the membrane fluorescence varied significantly. This can be partially ascribed to positioning out of the focal plane or focus instabilities during image acquisition between channels (tiny focus drift), but the potentially nonuniform distribution of the lipid dye and TCNQ should not be discounted either.

**Figure 2:**
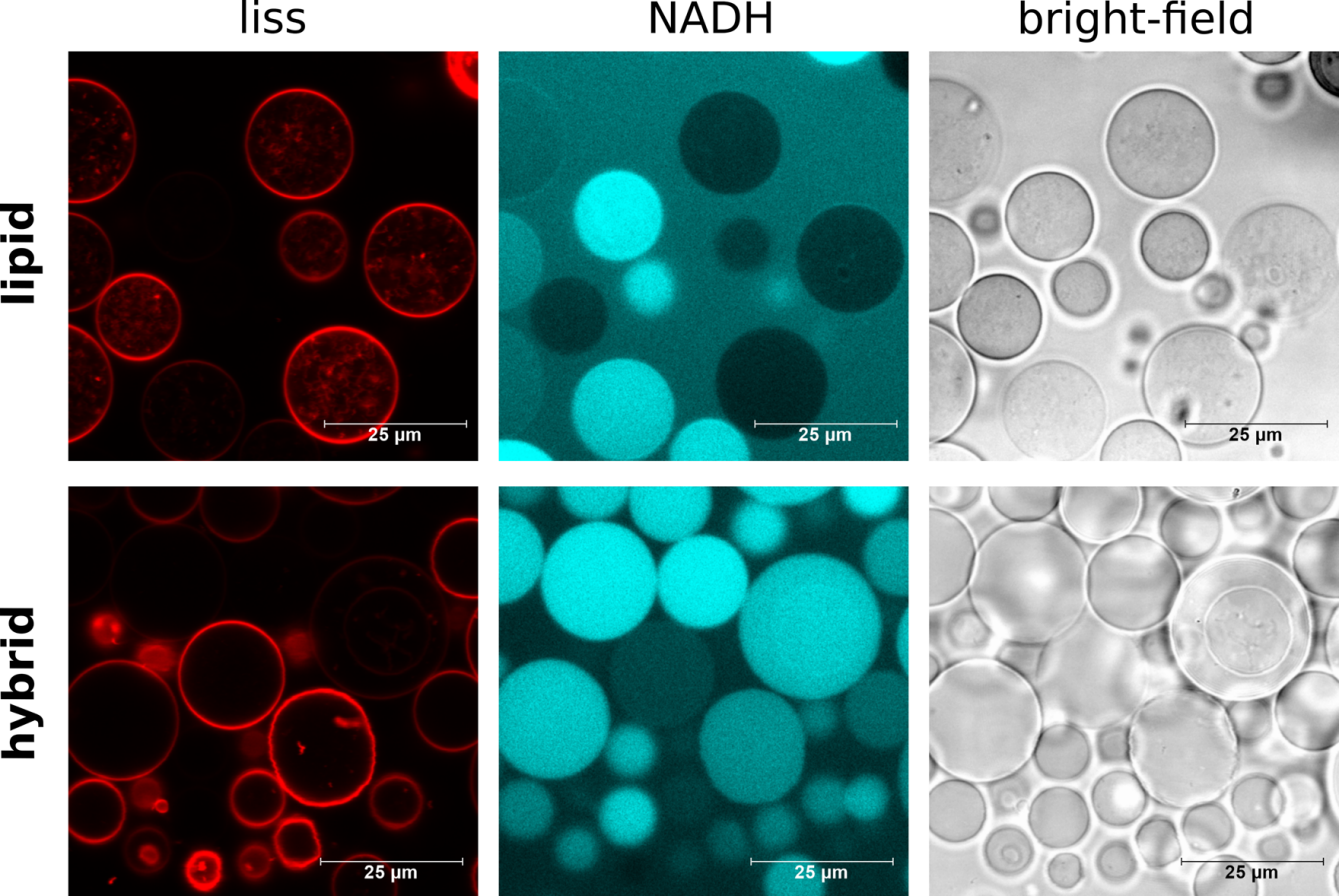
Confocal images of 100 mol % POPC GUVs (upper panel) and 20 mol % POPC/80 mol % PDMS-*g*-PEO GUVs (lower panel) with embedded TCNQ and encapsulated NADH. Left: liss-labeled membrane (red); middle: encapsulated NADH (cyan); right: bright-field channel. The red signal associated with the lipid vesicles (perceived as interior) is due to membrane protrusions.

CLSM indeed allowed monitoring of the NADH fluorescence, and the transmembrane oxidation proceeded faster (within a few minutes) compared to the nanometer scale due to the different ratios of ferricyanide to NADH. The amount of GUV-encapsulated cofactor (1 mM) was limited by the experimental procedure, and we employed higher oxidant concentration (10 mM) to accelerate the process and avoid long imaging. However, the resulting narrow time window for locating the GUVs and adjustment of imaging parameters prevented the analysis of sufficient individual events, whereby lowering the oxidant concentration twice did not solve this issue. Altogether, the mass transport could not be controlled during pipetting of ferricyanide aliquots on the observation slide. This resulted in the exposure of the compartments to different ferricyanide concentrations at different times. Moreover, bleaching of NADH hampered kinetic quantification also in CLSM (although not as pronounced as with standard epifluorescence) by the inability to discriminate it from past oxidation events.

In some cases, we noticed that the spherical membrane assumed an irregular shape upon full NADH oxidation (Figure S5), which indicated adsorption of the GUV to the glass surface (deflation was unlikely due to the matching osmolarity). Vesicle fusion to surfaces is known to be favored by lower pH and high ionic strength.^25^ In fact, upon direct oxidation of 1 mM NADH by a 20-fold excess of ferricyanide in bulk, the pH decreased from 5.5 to 4.2, and therefore, the observed adsorption was ascribed to acidification of the unbuffered solution. This experimental artifact actually demonstrates that a pH gradient can be achieved merely by the liberation of protons in the reaction, as previously shown with iron–sulfur peptide catalysts.^2^

To quantify the mediated NADH oxidation in GUVs, we made use of the higher signal-to-noise ratio of a novel time-correlated single-photon counting camera under very low illumination, which was developed for wide-field fluorescence lifetime imaging (FLIM).^26^ The background photons during the experiment, acquired by blocking the laser, were below 60 per second. A similar setup was previously used to unveil the desynchronization of glycolytic oscillations in yeast.^27,28^ The short time window after ferricyanide addition made it difficult to follow enough hybrid GUVs and prevented their analysis, but quantification of lipid GUVs was successful due to their slower transmembrane electron transfer kinetics. The time-resolved fluorescence of individual lipid vesicles varied with respect to the initial intensity (Figure 3a), which was due to the combination of different encapsulations and the integral photon detection (larger GUVs emitted more photons in sum, as evidenced by a positive linear correlation with the diameter: ρ = 0.76, Figure S6). Nevertheless, the vast majority of the compartments exhibited a common sigmoidal profile with a varying onset (Figure S7) upon addition of oxidant, while the GUVs lacking the mediator were unresponsive as expected (normalized traces without and with TCNQ in Figures 3b and 3c).

**Figure 3:**
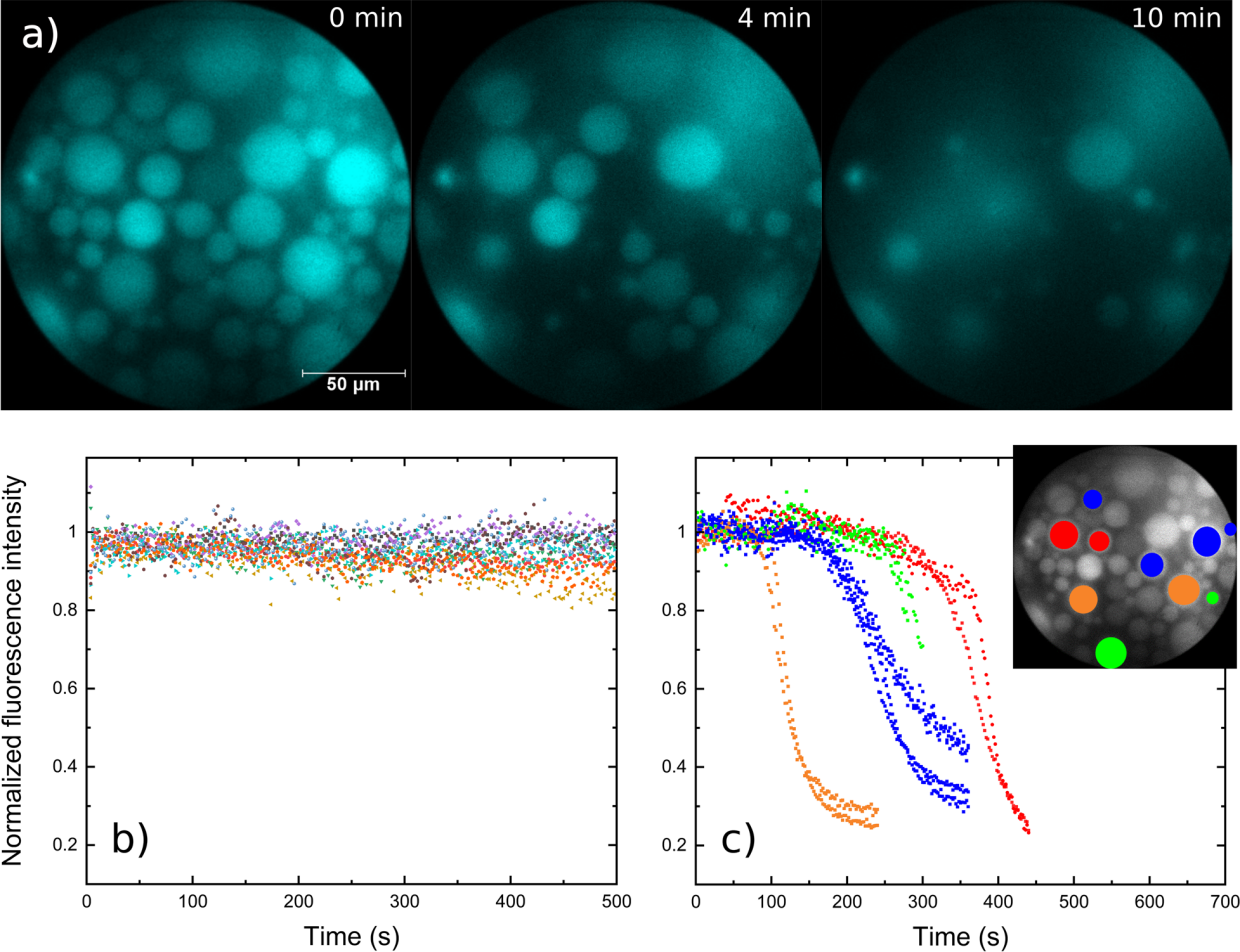
A) Time lapse of NADH oxidation in lipid (100% POPC) GUVs with embedded TCNQ monitored by time-correlated single photon counting (TCSPC) wide-field microscopy. Approximate time upon addition of ferricyanide is indicated in the upper right. B) Normalized NADH fluorescence of GUVs without TCNQ in the presence of ferricyanide. C) Selected profiles of the normalized NADH fluorescence of GUVs with embedded TCNQ in the presence of ferricyanide. Several traces are grouped by onset and designated by color code (orange, blue, green, red). Inset shows the respective position of the GUVs with the same color code.

The mild excitation by short laser pulses (averaged laser power <5 mW cm^–2^) resulted in negligible bleaching. This was tested before the addition of ferricyanide over several hours (time course within test window in Figure S8). The stability of the NADH signal allowed for extraction of the lumped electron transfer rate constants by following a manual workflow. Toward this end, the fluorescence intensity before the decrease was averaged, and the value was used to normalize the respective signal for more convenient comparison (Figure S9). The fluorescence profiles were then fitted by an exponential function, starting from the highest decrease rate, and GUVs that did not exhibit a clearly defined sigmoidal profile (<30%) were excluded. This resulted in an apparent electron transfer rate constant of 2.8 ± 1.8 × 10^–2^ s^–1^ for POPC GUVs. The slower decrease at the beginning and the delayed onsets of NADH oxidation were ascribed to the highly irregular ferricyanide diffusion front (onsets could not be correlated to the position of the GUVs, Figure 3c).

Unlike the bulk analysis at the nanoscale, which determines the behavior of the entire population, monitoring individual GUVs may provide a finer level of detail. In fact, observation of single nanovesicles has been reported several times, e.g., for deeper analysis of proton permeation by total internal reflection fluorescence (TIRF) microscopy.^29^ However, the optical access at the microscale potentially enables the correlation of activity with additional factors such as size, number of lamellae, membrane protrusions, etc. For this reason though, the ferricyanide supply would need to be controlled, e.g., by the use of microfluidic devices.^30^

The NAD(P)/NAD(P)H ratio is a hallmark for many cellular processes, and the NAD(P)H signal is widely used as a readout for enzymatic fluorescent assays. Precise NADH analysis is required for the design of minimal systems in the context of bottom-up synthetic biology as well.^31,32^ In the present study, we showed that synthetic augmentation may improve the transmembrane electron transfer by introducing favorable membrane properties, while the scaling up to micrometer vesicles provided an unequivocal demonstration of the process at biologically relevant dimensions and NADH concentrations. Thereby, the analysis of the membrane and oxidation kinetics required the use of CLSM and time-correlated single photon counting (TCSPC) wide-field microscopy. The latter imaging method accounts for sample heterogeneity and can be easily extrapolated to the investigation of passive or facilitated membrane transport, when using other sensitive fluorophores.

## Supporting Information Available

Materials and methods; size distribution of LUVs (DLS); fluorescent/UV–vis spectrophotometric data for electron transfer/ferricyanide leakage; epifluorescence/confocal images of GUVs; TCSPC-based fluorescence profiles of GUVs; and workflow for extraction of electron transfer rate constants (PDF)
