# Supplementary material for: Scale up of Transmembrane NADH Oxidation in Synthetic Giant Vesicles
Source: Bioconjug Chem. 2021 Apr 27;32(5):897–903. doi: 10.1021/acs.bioconjchem.1c00096 (PMC8154200; doi:10.1021/acs.bioconjchem.1c00096)
Supplement: Supplementary file 1 — bc1c00096_si_001.pdf [file bc1c00096_si_001.pdf]

Supporting Information

Scale up of transmembrane NADH oxidation in synthetic giant vesicles

MinHui Wang[†], André Weber[‡], Roland Hartig[§], Yiran Zheng[†], Dorothee Krafft[†], Tanja Vidaković-Koch[^], Werner Zuschratter[‡], Ivan Ivanov[†],* Kai Sundmacher[†,∇]

[†]*Process Systems Engineering, Max Planck Institute for Dynamics of Complex Technical Systems, Sandtorstrasse 1, 39106 Magdeburg, Germany*

[‡]*Combinatorial Neuroimaging Core Facility, Leibniz Institute for Neurobiology, Brenneckestrasse 6, 39118 Magdeburg, Germany*

[§]*Institute of Molecular and Clinical Immunology, Otto-von-Guericke University Magdeburg, Leipziger Strasse 44, 39120 Magdeburg, Germany*

[^]*Electrochemical Energy Conversion, Max Planck Institute for Dynamics of Complex Technical Systems, Sandtorstrasse 1, 39106 Magdeburg, Germany*

[∇]*Department of Process Systems Engineering, Otto-von-Guericke University Magdeburg, Universitätsplatz 2, 39106 Magdeburg, Germany*

*corresponding author: ivanov@mpi-magdeburg.mpg.de

Experimental details

Materials

1-palmitoyl-2-oleoyl-*sn*-glycero-3-phosphatidylcholine (POPC) and 1,2-dioleoyl-*sn*-glycero-3-phosphoethanolamine-N-(lissamine rhodamine B sulfonyl) (ammonium salt) (Liss) dissolved in chloroform were purchased from Avanti Polar Lipids Inc. (Alabaster, AL, USA). Reduced nicotinamide adenine dinucleotide (NAH) disodium salt was purchased from Carl Roth GmbH (Kalsruhe, Germany). 7,7,8,8-tetracyanoquinodimethane (TCNQ) was purchased from Sigma-Aldrich Chemie GmbH (Steinheim, Germany). Potassium ferricyanide was purchased from Merck KGaA (Darmstadt, Germany). Poly(dimethyl siloxane)-graft-poly(ethylene oxide) (PDMS-*g*-PEO, MW

3000 g mol^{-1} according to manufacturer) was a kind gift from Dow Corning Inc. (Midland, MI, USA). Distilled water was obtained from a Milli-Q water purification system (Millipore Corp.). All other chemicals were of analytic grade quality and used without further purification.

Preparation and characterization of large unilamellar vesicles (LUVs)

LUVs of different lipid/polymer molar ratios (POPC:PDMS-*g*-PEO = 0:100, 20:80, 50:50, 80:20, 100:0) were prepared by dry film hydration and subsequent extrusion. In each glass vial, the total amount of amphiphiles dissolved in chloroform was adjusted to 5 µmol. In cases when TCNQ was reconstituted, 66 µL of 0.31 mg mL^{-1} TCNQ solution in acetonitrile was added, corresponding to a molar ratio of amphiphiles to TCNQ 50:1. Afterwards the mixtures were dried under gentle N_2 flow and vacuum for 2 h to remove the solvents and then rehydrated by adding 500 µL of 10 mM NADH dissolved in MOPS buffer (100 mM MOPS, 250 mM KCl, Tris, pH 7.2). To detach the thin film, the hydration solution was gently pipetted against the walls of the vials multiple times and vortexed to resuspend homogeneously. Then the suspensions were subjected to six freeze/thaw cycles, where each cycle consisted of freezing in liquid N_2 for 1–2 min and thawing for 2–4 min in 40 ^{o}C water bath. In the last step, the suspensions were extruded 11 times through a Nuclepore Track-Etch Membrane filter (0.2 µm pore size, Whatman PLC, UK). After extrusion, the non-encapsulated NADH was separated via a disposable PD-10 desalting column (GE Healthcare Life Sciences) filled with 8.3 mL Sephadex G-25 matrix (Ø 14.5 mm × ca. 50 mm) and the purified vesicles were collected and stored at 4 ^{o}C for later use.

The size of the LUVs was determined by dynamic light scattering (DLS) using a Zetasizer Nano ZS (Malvern, Worcestershire, UK) with a 663 nm HeNe laser at a fixed scattering angle of 173° at room temperature. Every measurement was run three times with 70 s duration of each single run and the hydrodynamic diameter was reported as the average.

The transmembrane oxidation was assessed by monitoring the fluorescence intensity of NADH on a Cary Eclipse Fluorescence Spectrophotometer (Agilent Technologies, Santa Clara, USA). The respective vesicle suspensions were transferred to a 10 mm quartz cuvette (500 µL, Hellma, Germany) and the fluorescence was monitored at 460 nm (slit width: 20 nm) with excitation wavelength of 340 nm (slit width: 20 nm), prior and upon addition of 400 µM potassium ferricyanide.

Preparation and characterization of giant unilamellar vesicles (GUVs)

GUVs composed of POPC and POPC/PDMS-*g*-PEO = 20:80 were prepared by conventional electroformation. Briefly, 25 µL of 1 mg ml^{-1} amphiphilic molecules dissolved in chloroform without and with TCNQ (at the same ratio as in LUV experiments) were spread on two glass slides coated with indium tin oxide (ITO, resistivity 20 Ω/sq). To better visualize the GUV membrane, 0.05 mol% of Liss was added to the amphiphile mixture. The solvent was evaporated under N_2 and after placing 2-mm-thick silicone spacer, the paired glass slides were fixed with a clip and filled with 1 mM NADH solution dissolved in 0.2 M sucrose in Milli-Q water. During the electroformation, a 10 Hz sine wave with 2 V amplitude was applied for 45 min, followed by a 2 Hz squared wave with 1 V amplitude for 15 min. Afterwards GUVs were gently collected and stored at 4 ^{o}C for later use. Before imaging, a three-fold volume of glucose solution, whose osmolarity was matched to the inner sucrose solution, was added to the vesicle suspensions. The time course of NADH oxidation was followed upon addition of potassium ferricyanide to a final concentration of 10 mM. The osmolarity values were measured with Osmomat 3000 (Gonotech GmbH, Berlin, Germany).

GUVs were analyzed using conventional epifluorescence microscopy (FM), confocal laser scanning microscopy (CLSM) and time-correlated single photon counting (TCSPC) wide-field microscopy. The FM apparatus consisted of a Zeiss inverted microscope (Zeiss, Jena, Germany), equipped with mercury (HBO) arc lamps and an AxioCam 506 color camera. Liss (Excitation: 560 nm, Emission: 570–640 nm) and encapsulated NADH (Excitation: 340 nm, Emission: 420–460 nm) were detected. Images were processed using Zen software (Zeiss). CLSM was performed using a SP8 inverted microscope system (Leica, Mannheim, Germany). Images (Excitation: 355 nm, Emission: 400–800 nm) were obtained and processed using a 63×/1.4 oil immersion objective and LAS X software (Leica), after which time lapse images were extracted. To characterize the dynamics of NADH oxidation, the sensitivity of time-correlated single photon counting technique was used in a wide-field microscope system consisting of a Nikon TiEclipse microscope (Nikon GmbH, Düsseldorf, Germany) and a white light laser (NKT Photonics, Denmark) in combination with a SuperK Extend-UV module (NKT Photonics, Denmark). The laser was tuned to 355 nm and light pulses with a repetition rate of 19.5 MHz and 40 ps pulse width (FWHM) were generated. The collimated laser pulses were focused on the back focal plane of the UV-transparent objective (100× S-Fluor,

NA 1.3, Nikon GmbH, Düsseldorf, Germany) to produce an uniformly illuminated field of view. A dichroic mirror (LP 355 nm, AHF, Tübingen, Germany) was used to discriminate between the excitation and the fluorescence signal. The emitted light was filtered by bandpass filter (BL 447/60, AHF, Tübingen, Germany). The fluorescence signal collected at the objective was projected on a sensitive wide-field camera based on a position-maintaining microchannel plate photomultiplier tube (MCP-PMT) with a direct charge readout of the position and an electronic interface for the data transfer (LINCam25; Photonscore Magdeburg, Germany). For image analysis, the incident single photons were binned to a 2D histogram according to their space coordinates of 256×256 pixel for time intervals of 2 s. The resulting videos were processed with ImageJ Fiji freeware.

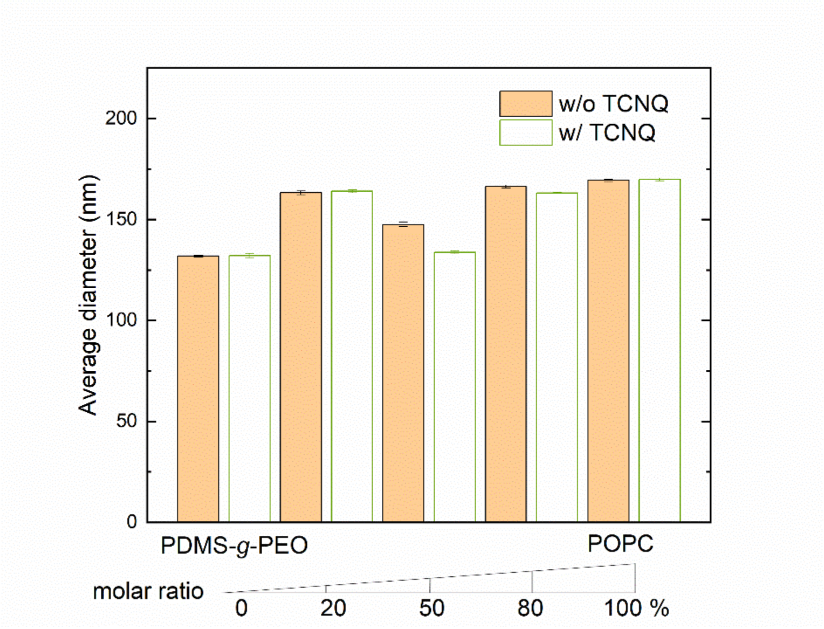

Figure S1: Average diameters (DLS) of vesicles with different molar ratio of POPC and PDMS-*g*-PEO with and without incorporated TCNQ.

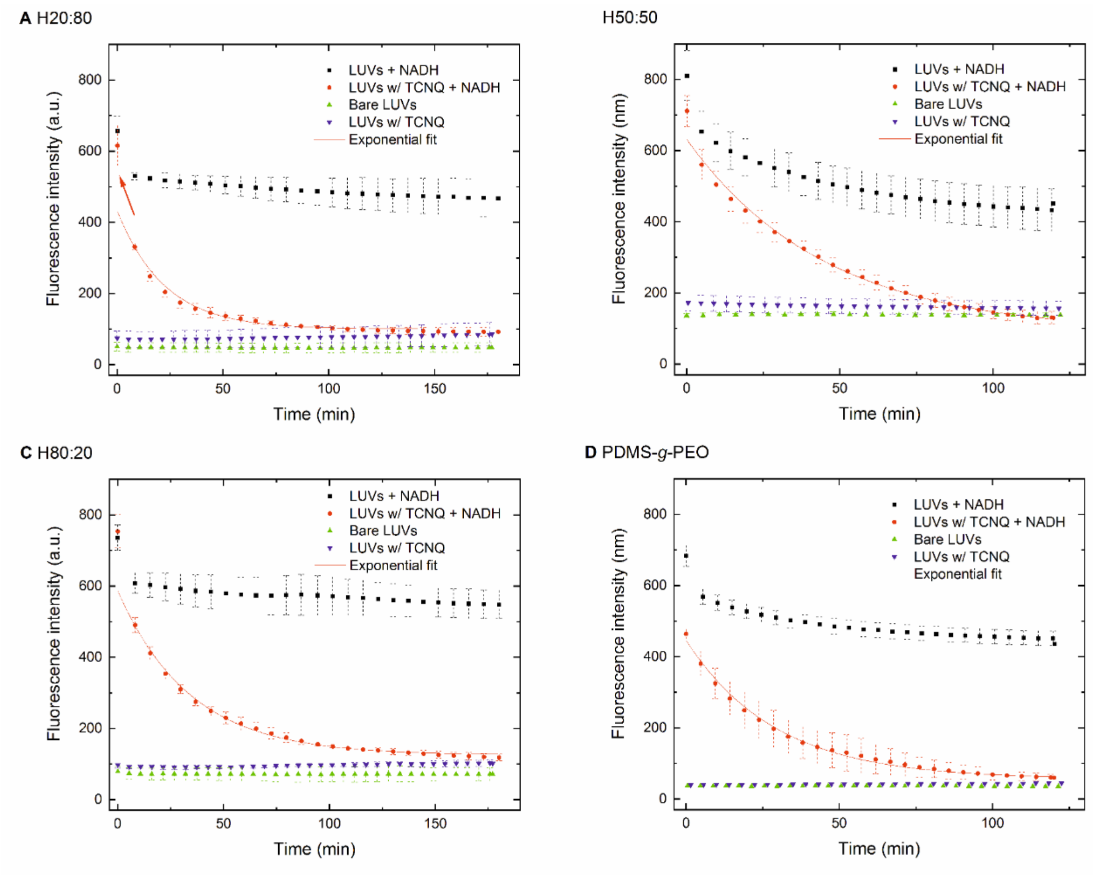

Figure S2: Fluorescence intensity profiles (spectrophotometric) of bare and functionalized vesicles of different compositions in absence (blue and green symbols) and presence (black and red symbols) of 400 µM potassium ferricyanide: A) 20 mol% POPC, 80 mol% PDMS-g-PEO; B) 50 mol% POPC, 50 mol% PDMS-g-PEO; C) 80 mol% POPC, 20 mol% PDMS-g-PEO; D) 100 mol% PDMS-g-PEO.

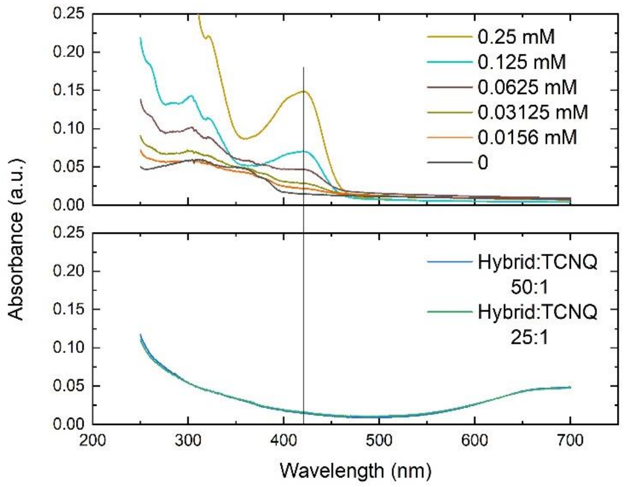

Figure S3: UV-Vis absorbance spectra of different potassium ferricyanide concentrations (upper panel) and of hybrid vesicles composed of 20 mol% POPC and 80 mol% PDMS-g-PEO and two molar ratios of incorporated TCNQ after incubation and gel filtration (lower panel). The ratio of the total volume of vesicles and the outer solution is about 1:100.

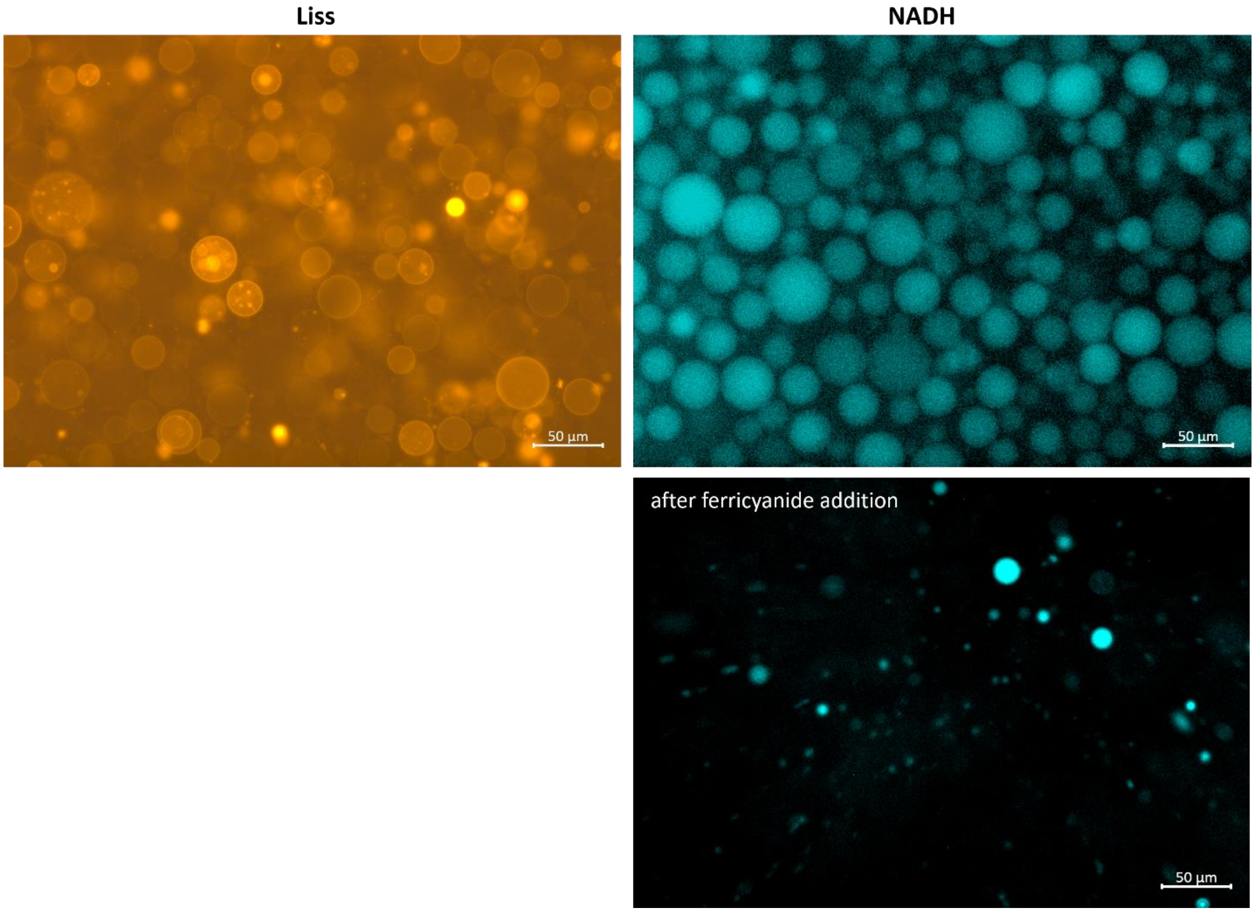

Figure S4: Fluorescence images (standard epifluorescence microscopy) of hybrid GUVs (20 mol% POPC, 80 mol% PDMS-g-PEO) with embedded TCNQ and encapsulated NADH. Left: membrane labeled with DOPE-N-(Lissamine rhodamine B sulfonyl), yellow; right: NADH (1 mM NADH in 0.2 M sucrose was used for electroformation and the resulting GUV suspension was diluted three-fold with isosmotic glucose solution), cyan. GUV suspension upon addition of 10 mM ferricyanide shown below.

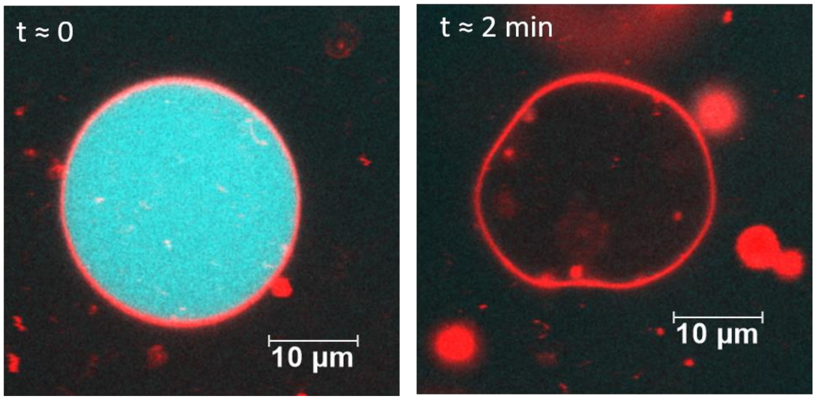

Figure S5: Merged (Liss: red and NADH: cyan) fluorescence images from confocal laser scanning microscopy of a hybrid GUV with embedded TCNQ shortly after NADH oxidation onset. Approximate time upon addition of ferricyanide is indicated in the upper left.

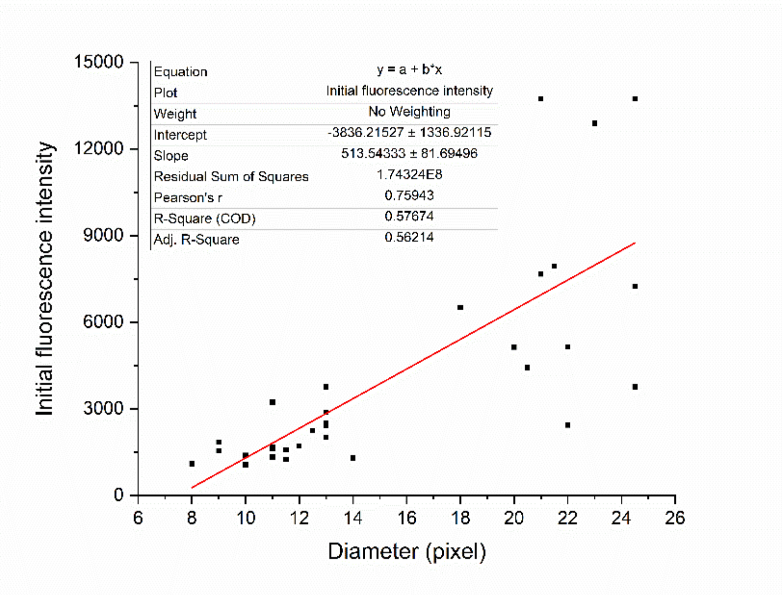

Figure S6: Correlation between the initial intensity of POPC GUVs (NADH fluorescence) with incorporated TCNQ and their diameter detected by time-correlated single photon counting wide-field microscopy. Red line shows the linear fitting of all points. Fitting report shown as inset.

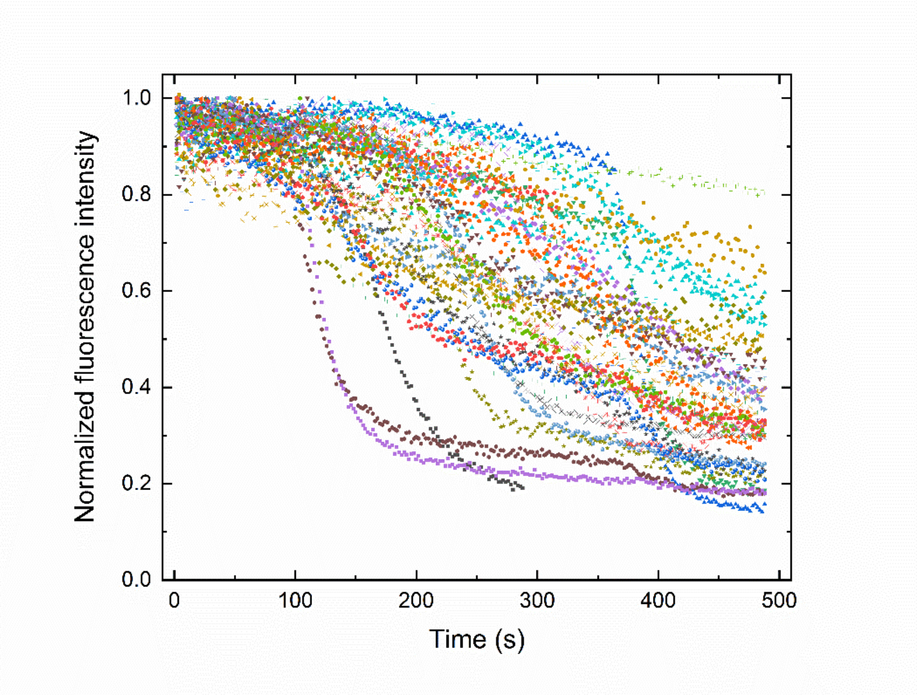

Figure S7: Time courses of normalized NADH fluorescence intensity of lipid GUVs with incorporated TCNQ in presence of ferricyanide monitored by time-correlated single photon counting wide-field microscopy.

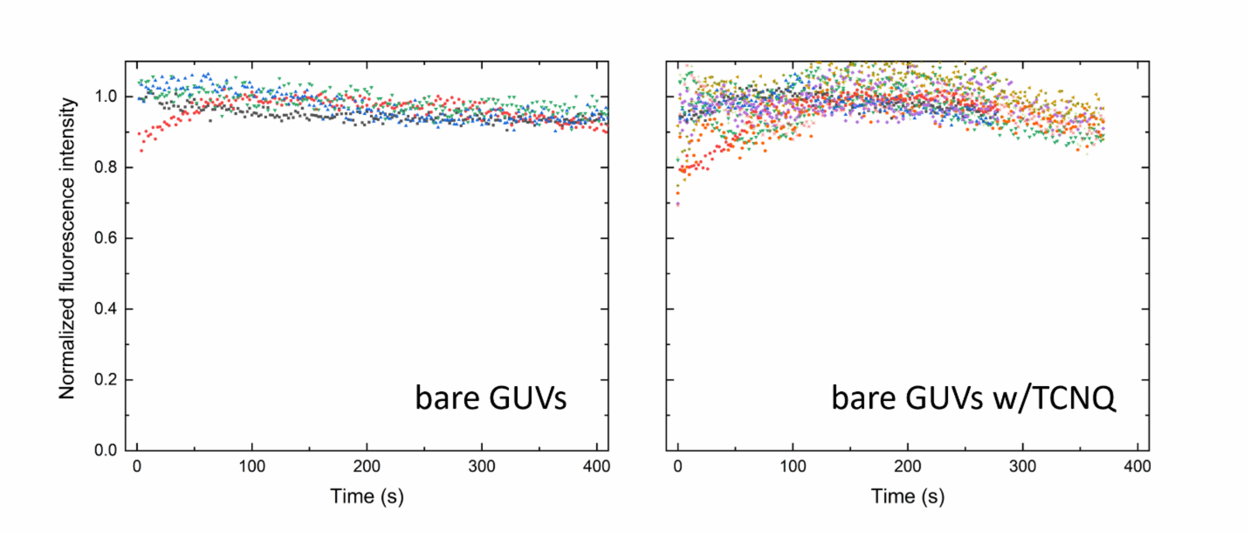

Figure S8: Time courses of normalized NADH fluorescence intensity of lipid GUVs without (left) and with incorporated TCNQ (right) monitored by time-correlated single photon counting wide-field microscopy.

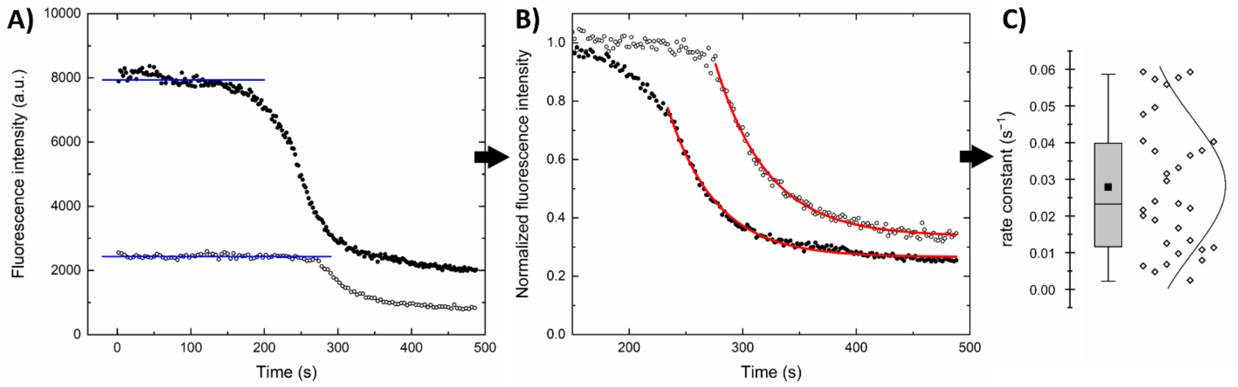

Figure S9: Analysis workflow for extraction of rate constants from time-correlated single photon counting wide-field microscopy. A) Two representative examples with higher and lower initial intensity, blue lines indicate averaged initial intensities; B) Normalized traces and exponential fitting curves, red curves; C) box plot and normal distribution of the rate constants (n=32).
